# How do teachers’ roles transform in metaverse classroom? The mechanisms of awe experience and differences across teaching stages

**DOI:** 10.3389/fpsyg.2025.1663964

**Published:** 2026-01-12

**Authors:** Hanxi Li, Yuanxiong Liu, Jiang Du, Younghwan Pan

**Affiliations:** 1 Faculty of Art and Communication, Kunming University of Science and Technology, Kunming, China; 2 Department of Smart Experience Design, Kookmin University, Seoul, Republic of Korea

**Keywords:** awe experience, metaverse classroom, multi-group analysis, PLS-SEM, role clarity, teaching engagement, teaching satisfaction

## Abstract

With the rapid development of metaverse technologies in education, how teachers adapt to this emerging environment has become a critical issue. This study explores teachers’ emotional experiences and adaptation mechanisms within metaverse classroom. Grounded in awe experience theory and role theory, it examines how classroom features influence teachers’ role adaptation through both positive and negative awe experiences, thereby affecting teaching satisfaction and engagement. Using survey data from 692 primary and secondary teachers, Partial Least Squares Structural Equation Modeling (PLS-SEM) and Multi-Group Analysis (MGA) were conducted. Results show that the immersive, interactive, technically complex, and decentralized metaverse environments significantly enhance positive awe while unexpectedly reducing negative awe. These emotional responses improve teachers’ role clarity, leading to higher teaching satisfaction and engagement. Moreover, Multi-group analysis reveals stage-based differences: primary school teachers are more easily inspired by positive awe, whereas high school teachers demonstrate stronger technological adaptability and emotional regulation. This study not only expands the understanding of emotional mechanisms among teachers in educational technology research but also provides stage-specific and differentiated practical implications for teacher training and instructional design.

## 1 Introduction

With the rapid advancement of digital technologies, immersive technologies such as virtual reality (VR) and augmented reality (AR) have become central to the development and application of the metaverse in education. The metaverse classroom breaks the physical boundaries of traditional teaching by providing immersive interactive experiences and virtual learning environments, offering teachers and students unprecedented teaching and learning experiences (Di Natale et al., 2024). At present, metaverse classroom have been widely implemented from primary to higher education around the world, significantly transforming conventional teaching methods and learning behaviors (Koo, 2021; Tlili et al., 2022). In China, the development of metaverse-based education has also received strong policy support and practical exploration. In February 2024, the Ministry of Education announced 184 artificial intelligence education pilot schools at the primary and secondary levels, encouraging the deep integration and routine application of emerging technologies such as VR and AR in future classrooms.

Despite the growing body of research focusing on the technological infrastructure of metaverse classroom, teaching methods, and their impact on student learning outcomes, limited attention has been given to how teachers adapt and transform within this emerging educational environment. In metaverse classroom, the role of teachers is gradually shifting from traditional knowledge transmitters to facilitators and collaborators in the learning process (Hwang and Lee, 2024). This transformation involves not only changes in instructional methods but also deeper psychological adaptation and redefinition of teachers’ role perceptions. During this transition, teachers may experience complex emotional responses. On the one hand, the sense of immersion and interactivity brought by new technologies can stimulate positive emotions such as creativity and enthusiasm for teaching. On the other hand, the uncertainty introduced by these technologies and the redistribution of instructional control may also lead to negative emotions such as anxiety and role ambiguity (Henderson and Corry, 2021). Therefore, it is essential to explore teachers’ emotional experiences and role adaptation mechanisms in the context of metaverse classroom.

Against this background, awe experience as an emotional response that integrates surprise, reverence, and cognitive expansion has become a key psychological mechanism for teachers to understand and adapt to the metaverse classroom environment. In addition, role clarity which refers to teachers’ clear understanding of their responsibilities, tasks, and expectations is a critical factor in adapting to new roles and achieving psychological adjustment during the teaching process (Hassan, 2013; Kauppila, 2014). It also plays an important role in helping teachers adapt to new technological environments. Therefore, incorporating awe experience and role clarity into research on teachers’ adaptation to metaverse classroom not only provides deeper insights into their psychological adjustment mechanisms but also offers new theoretical and practical perspectives for understanding teachers’ role transformation.

Moreover, teachers at different educational stages such as primary, junior high, and senior high school may exhibit distinct patterns of role adaptation due to differences in student groups, teaching objectives, and educational pressures. Previous studies have rarely examined these stage-specific differences among teachers, which limits a comprehensive understanding of their adaptation mechanisms. Therefore, this study adopts a group analysis approach to explore differences in emotional experiences and role adaptation among teachers at different educational stages within the metaverse classroom, aiming to provide targeted guidance for teacher training and support.

In summary, this study aims to address the following research questions: (1) Which features of the metaverse classroom significantly influence teachers’ positive and negative awe experiences? (2) How do positive and negative awe experiences affect teachers’ role clarity? (3) How does role clarity further influence teaching satisfaction and teaching engagement? (4) Do teachers at different educational stages show significant differences in emotional experience and role adaptation pathways? By exploring these questions, this study seeks to clarify the mechanism through which awe experience contributes to teachers’ role adaptation and to provide both theoretical foundations and practical suggestions for the educational application of metaverse classroom.

## 2 Literature review and hypotheses

### 2.1 Metaverse classroom

The metaverse classroom provides teachers and students with a virtual educational space for real-time interaction and collaboration through highly realistic virtual environments (Alfaisal et al., 2024). Unlike traditional classrooms, online learning, or general virtual classrooms, the metaverse classroom emphasizes a deep integration of virtual and physical realities, creating an educational ecosystem that transcends physical limitations while maintaining a sense of realism for users (Pradana and Elisa, 2023; Tlili et al., 2022). For example, physical constraints and resource limitations in traditional classrooms can be overcome through metaverse technologies, allowing students to participate in learning activities and access educational resources anytime and anywhere. In addition, the metaverse classroom offers personalized and interactive learning experiences, going beyond the limitations of traditional online education which often lacks real interaction and emotional connection (Sarıtaş and Topraklıkoğlu, 2022).

Existing research has identified several core features of the metaverse classroom: Immersion enables students to experience virtual environments as if they were physically present, thereby enhancing their understanding and absorption of abstract concepts. Many studies have shown that immersion can effectively improve student engagement and concentration, leading to better learning outcomes (Petersen et al., 2022). Interactivity refers to the real-time communication and interaction between students and teachers, peers, or virtual objects in the virtual space (Vercellotti, 2018). For example, through virtual chemistry experiments or the reconstruction of historical scenes, students can intuitively experience the advantages of hands-on learning and situational instruction. In addition, the metaverse classroom involves a high level of complexity. Although the rich technical operations and diverse learning content offer abundant resources, they also increase the technological burden and cognitive load for both teachers and students (Brown, 2015). Decentralization is reflected in the shift of the teacher’s role from knowledge transmitter to facilitator and collaborator, encouraging students to participate more actively in the teaching process (Polychronaki et al., 2024). The metaverse classroom also features real-time feedback, personalized learning paths, and user co-creation (Al-kfairy et al., 2024).

At present, the application of metaverse classroom in higher education has achieved initial progress. Scholars from various countries have actively explored its technical feasibility, user acceptance, and platform design (Alkhwaldi, 2023; Grover et al., 2023; Rodriguez-Florido et al., 2024). However, research focusing on teachers remains relatively limited. Existing studies have pointed out that in technology-enhanced classroom environments, teachers often face negative effects such as technological anxiety and excessive content load. The mere introduction of new technologies does not guarantee improvements in teaching outcomes (Vázquez-Cano et al., 2023; Waquar et al., 2024). Although metaverse classroom provide abundant teaching possibilities, they also place higher demands on teachers’ adaptability. Teachers need not only to master the use of new technological tools but also to conceptualize the organization and presentation of course content and to balance instructional control and facilitation during interactive processes.

Therefore, understanding how teachers construct internal mechanisms to adapt to complex teaching environments—including their cognitive tendencies, coping strategies, and behavioral patterns when facing novel teaching scenarios—has become a key factor in promoting the sustainable development of educational digital transformation. In summary, in-depth research on teachers’ role adaptation and psychological transformation in metaverse classroom can effectively promote the deep integration of metaverse technologies and educational practice and support the advancement of education toward higher quality and greater sustainability.

### 2.2 Awe experience theory

The concept of awe originally emerged in the fields of philosophy and religion to describe a complex emotional experience that arises when individuals encounter vast, grand, or inexplicable phenomena (Keltner and Haidt, 2003). In recent years, the psychology field has conducted systematic explorations of awe experiences. Keltner and Haidt (2003) emphasized two core components of awe. The first is perceived vastness, referring to the sense of spatial, temporal, or social magnitude and complexity. The second is the need for accommodation, meaning the individual’s need to adjust existing cognitive frameworks to comprehend new and unfamiliar stimuli (Shiota et al., 2007).

From the perspective of psychological mechanisms, awe experience stems from the interaction between cognitive uncertainty and the need for cognitive adaptation in response to the external environment. When individuals face complex phenomena that cannot be explained by their current knowledge, their internal psychological balance is disrupted, leading to feelings of confusion and a diminished sense of self. This disruption stimulates an intrinsic motivation for exploration, learning, and restructuring of cognitive schemas (Shiota et al., 2007; Gordon et al., 2017). During this process, awe involves a perceived reduction of the self, prompting individuals to see themselves as part of a greater whole. This experience of the small self fosters deeper cognitive engagement with and understanding of the surrounding environment (Bai et al., 2017).

In terms of cognitive influence, awe experience can significantly alter individuals’ cognitive structures and thinking patterns. Studies have found that awe encourages individuals to confront uncertainty and ambiguity with greater openness, enhancing creative thinking, cognitive flexibility, and receptiveness to new experiences (Shiota et al., 2007). Moreover, awe is considered a cognitive-based emotion that plays a critical role in promoting deep learning and conceptual change. In complex contexts or emerging knowledge domains, individuals are more likely to experience cognitive and behavioral shifts after undergoing awe experiences (Gail Jones et al., 2022).

Awe experience can be divided into two dimensions positive awe and negative awe. Positive awe is typically associated with feelings of pleasure, beauty, or wisdom and is characterized by inspiration, expansiveness, and wonder (Tong et al., 2025). For example, magnificent natural landscapes, grand artistic works, or great scientific discoveries may evoke positive awe, leading individuals to feel inspired, engage in creative thinking, and develop a deeper understanding of the world (Shiota et al., 2007; Gail Jones et al., 2022). Negative awe, on the other hand, is marked by feelings of being overwhelmed, anxious, insignificant, and out of control. It often occurs when individuals face unknown or highly complex situations beyond their control such as major natural disasters or advanced emerging technologies which may trigger heightened psychological stress and discomfort (Sawada and Nomura, 2020).

At present, awe experience has been widely applied across various fields including psychology, consumer behavior, and organizational behavior. In psychology, studies have shown that awe can effectively promote prosocial behavior and enhance individuals’ sense of connection with others and their environment (Piff et al., 2015). In the field of consumer behavior, awe experience has been used to analyze how consumers derive emotional satisfaction from the grand narratives of brands or products, which in turn influences their decision-making processes (Cavanaugh, 2025). In organizational behavior research, awe experience has been found to enhance employees’ sense of identification with their organization and increase their work engagement, thereby improving job satisfaction and performance (Sheprow and Harrison, 2022). Research in new technology environments also suggests that emerging technologies such as virtual reality VR and augmented reality AR possess unique immersive and interactive features that can effectively evoke users’ feelings of awe and subsequently enhance immersion and cognitive engagement (Quesnel and Riecke, 2018; Zhao et al., 2025).

In the field of education, existing research on awe experience has predominantly focused on students. Prior studies have demonstrated that awe can enhance students’ learning interest and motivation, and facilitate deeper understanding of complex knowledge (Gail Jones et al., 2022). However, teachers—as key agents in the implementation of educational technologies—have received comparatively limited attention, particularly regarding their awe experiences and psychological adaptation mechanisms in emerging technology-enhanced teaching environments. According to Keltner and Haidt’s framework, awe experience is jointly constituted by two core components: perceived vastness and the need for accommodation (Keltner and Haidt, 2003). In teaching contexts, these components are not abstract psychological constructs but are manifested through concrete instructional practices and interactions with technology. For instance, when teachers encounter immersive learning environments, multimodal interaction formats, or highly integrated instructional functions in metaverse classroom that transcend the boundaries of traditional classrooms, they may perceive an expansion of teaching space and pedagogical possibilities. Such perceptions challenge existing teaching experiences and require cognitive accommodation and restructuring.

Depending on teachers’ psychological interpretations, this challenge may be transformed into different forms of awe experience. On the one hand, when teachers interpret these expanded pedagogical demands as opportunities for professional growth and instructional innovation, awe is more likely to emerge in a positive form, characterized by inspiration, enhanced creative thinking, and proactive exploration of new teaching roles (Shiota et al., 2007). This form of positive awe is often accompanied by a renewed recognition of teaching potential and may support teachers in reconstructing their professional role perceptions within complex instructional environments. On the other hand, when teachers perceive that their existing abilities, experience, or resources are insufficient to cope with the high level of complexity introduced by the technological environment, the same demand for accommodation may elicit negative awe experiences. Such negative awe is typically manifested as feelings of overwhelm, confusion, and diminished perceived control over teaching processes, thereby posing challenges to teachers’ psychological stability and role cognition (Piff et al., 2015; Howard and Gigliotti, 2016).

Accordingly, teachers’ awe experiences in metaverse classroom exhibit a clear duality. Positive awe emphasizes an inspirational understanding of pedagogical possibilities and role expansion, whereas negative awe reflects uncertainty and perceived loss of control when teachers confront instructional situations that exceed established teaching paradigms. Based on this distinction, the present study conceptualizes teachers’ awe experience in metaverse classroom as comprising both positive and negative dimensions, and further examines their differential effects on teachers’ role clarity and teaching-related outcomes. In doing so, this study seeks to address the current gap in research on teachers’ emotional adaptation mechanisms in emerging educational technology contexts.

#### 2.2.1 Teaching immersion and awe experience

Immersion refers to the subjective feeling of being physically present in a virtual environment. It involves multisensory inputs such as visual, auditory, and tactile information, enabling individuals to enter a psychological state in which they feel detached from reality and fully engaged in the virtual context (Radianti et al., 2020; Conrad et al., 2024). According to psychological research, immersion is typically reflected in a user’s high level of concentration on the virtual environment, strong emotional involvement, and active cognitive engagement (Petersen et al., 2022). A high degree of immersive experience facilitates the shift from passive information reception to active knowledge construction, effectively enhancing learners’ motivation, cognitive depth, and learning performance (Conrad et al., 2024).

Recent studies have found that immersion can effectively induce awe experiences. Especially in VR environments, highly realistic visual scenes and interactive experiences greatly enhance users’ sense of telepresence, which can trigger strong feelings of awe (Chirico et al., 2017). For example, natural landscapes or magnificent scenes simulated through VR technology can significantly increase users’ perception of environmental vastness, thereby enhancing positive awe experiences (Kahn and Cargile, 2021). At the same time, excessive immersion may lead to anxiety or a sense of loss of control, thereby inducing negative awe experiences (Pizzolante et al., 2023).

As a highly immersive teaching environment that integrates advanced technologies, the metaverse classroom provides teachers with a novel educational setting. In this context, teachers may perceive the vastness of the virtual world, develop deeper cognitive insights into the teaching content, and engage in more creative thinking, which can trigger positive awe experiences. On the other hand, due to the complexity of the technological environment and the high level of realism in virtual experiences, teachers may feel increased cognitive load or even a loss of classroom control, which can lead to negative awe experiences. Therefore, this study proposes the following hypotheses:

*H1a:* Teaching immersion positively influences teachers’ positive awe experience.

*H1b:* Teaching immersion positively influences teachers’ negative awe experience.

#### 2.2.2 Teaching interactivity and awe experience

Interactivity generally refers to the degree of bidirectional communication between learners and teachers or instructional content during the teaching process. This interaction, achieved through real-time feedback, dynamic communication, and continuous engagement, can effectively enhance learners’ understanding and mastery of knowledge (Moore, 1989; Sandoval-Henríquez and Badilla-Quintana, 2021). Specifically, teaching interactivity goes beyond one-way information transmission and emphasizes interaction between teachers and students, between students and learning materials, and among students themselves. High levels of interactivity help create a positive learning atmosphere, increasing learner initiative and cognitive engagement (Johnson-Glenberg, 2018).

Previous studies have found that in VR environments, natural interactive methods such as gestures, movements, and verbal communication allow users to more deeply experience the realism of the environment, significantly enhancing the intensity of awe (Quesnel and Riecke, 2017). For example, in VR tourism experiences, users can explore and actively control the environment through interactive engagement, leading to a stronger sense of environmental vastness and personal insignificance, which effectively triggers positive awe experiences (Zhao et al., 2024). However, excessive interactivity and overly complex interaction mechanisms may also cause users to feel information overload or a lack of control, resulting in negative awe experiences (Pizzolante et al., 2023).

The teaching interactivity of the metaverse classroom presents richer forms and dimensions. Teachers can interact not only through speech and visual elements but also via gesture control, manipulation of virtual objects, and real-time collaboration with multiple users, achieving multimodal interaction (Gualano and Campbell, 2024). These interactive approaches help teachers better perceive instructional content within the virtual classroom, thus enhancing emotional engagement and fostering positive awe experiences. At the same time, high levels of interactivity may increase teachers’ technical burden and cognitive load, potentially causing stress toward new technologies and inducing negative awe experiences. Therefore, the following hypotheses are proposed:

*H2a*: Teaching interactivity positively influences teachers’ positive awe experience.

*H2b*: Teaching interactivity positively influences teachers’ negative awe experience.

#### 2.2.3 Technical complexity and awe experience

Technical complexity refers to teachers’ subjective perceptions of the operational demands, functional structure, and instructional integration difficulty when using metaverse classroom systems. Unlike traditional views that conceptualize technical complexity solely as a technological burden, recent studies have increasingly suggested that technical complexity in educational contexts may encompass both burdensome complexity and productive complexity (Tarafdar et al., 2007; Upadhyaya and Vrinda, 2021). On the one hand, when technological systems involve multilayered functions, complex interaction logic, or high learning costs, teachers are required to invest greater cognitive resources to understand and adapt to the system, which may increase psychological strain and perceived uncertainty. On the other hand, complex systems characterized by high functional integration and pedagogical potential may also be perceived as valuable resources for expanding instructional boundaries and reshaping teaching practices, thereby stimulating exploratory motivation and professional growth.

In immersive environments such as virtual reality (VR) or augmented reality (AR), technical complexity becomes particularly salient (Du et al., 2022). Teachers are not only required to become familiar with new devices and operational modes, but also to deeply integrate technological functions with instructional objectives, classroom pacing, and teacher–student interaction patterns. Such complexity may, on the one hand, elicit negative emotional responses such as anxiety and feelings of overwhelm; on the other hand, by revealing the scale, creativity, and pedagogical possibilities of technological systems, it may also evoke awe experiences among teachers—namely, a sense of wonder and reflection regarding the transformative potential of technology for teaching (Konigsburg, 2024; Quesnel and Riecke, 2018).

Within the context of metaverse classroom, teachers are confronted not only with operational complexity at the technological level, but also with the need to reconstruct their teaching roles and instructional practices. The teaching possibilities embedded in technical complexity—particularly those that extend beyond teachers’ prior instructional experiences—may give rise to positive awe experiences, such as inspiration and the expansion of pedagogical perspectives. At the same time, when teachers perceive a substantial gap between system demands and their own abilities or resources, technical complexity may also trigger negative awe experiences, manifested as diminished perceived control and heightened role uncertainty (Rezaeı et al., 2008; Chatzoglou et al., 2009; Joo et al., 2016). Based on this reasoning, the following hypotheses are proposed:

*H3a:* Technical complexity positively influences teachers’ positive awe experience.

*H3b:* Technical complexity positively influences teachers’ negative awe experience.

#### 2.2.4 Teaching decentralization and awe experience

Decentralization refers to an organizational or social structure that emphasizes the distribution of power, shared responsibility, and individual autonomy (Triantafillou, 2012). In traditional educational models, teachers usually occupy a dominant role in the classroom, being responsible for knowledge transmission, activity organization, and classroom management, while students tend to act as passive recipients of knowledge. In recent years, with the evolution of educational philosophy and the advancement of technological environments, educational practice has shown a clear trend toward decentralization. Accordingly, the role of the teacher has shifted from a transmitter of knowledge to a facilitator and collaborator in the learning process (Abdigapbarova and Zhiyenbayeva, 2023). This transformation has not only altered the power dynamics within classrooms but also significantly influenced the structure of teaching activities and the emotional experiences of both teachers and students (Bhardwaj et al., 2025).

In decentralized teaching environments, students often gain greater autonomy and more opportunities for exploration (Otto et al., 2024). Studies have shown that autonomy and exploratory freedom are key factors in eliciting awe experiences. For example, van Limpt-Broers et al. (2024) found that students exploring unknown cosmic spaces independently in virtual reality environments were more likely to experience awe, which enhanced their emotional engagement and cognitive focus. Similarly, Quesnel and Riecke (2018) observed that learners who independently selected content in virtual environments reported more intense awe experiences, showing greater emotional arousal and a sense of self-transcendence. These findings suggest that decentralized teaching environments can effectively stimulate awe by granting learners greater autonomy and exploratory opportunities.

In metaverse classroom, decentralization presents characteristics that differ significantly from those in traditional classrooms. Through highly interactive virtual avatars, teachers and students participate together in the teaching process, making instruction more equal, interactive, and collaborative (Singh et al., 2024). In this new educational context, teachers may experience positive awe that differs from traditional methods, such as admiration and appreciation for students’ growing autonomy and creative abilities. At the same time, the high degree of student autonomy and changing interaction patterns may also lead to feelings of reduced authority or a loss of classroom control, resulting in negative awe experiences. Therefore, this study proposes the following hypotheses:

*H4a:* Perceived decentralization in teaching positively influences teachers’ positive awe experience.

*H4b:* Perceived decentralization in teaching positively influences teachers’ negative awe experience.

### 2.3 Teachers’ role clarity

In metaverse classroom, teachers’ roles are gradually shifting from traditional knowledge transmitters to facilitators of learning processes and designers of instructional contexts (Singh et al., 2024). A critical condition for adapting to this transformation lies in whether teachers are able to develop clear role perceptions, namely role clarity, which refers to teachers’ explicit understanding of their responsibilities, task boundaries, and instructional expectations (Hassan, 2013). Prior research has shown that higher levels of role clarity are associated with increased teaching satisfaction and teaching engagement, while simultaneously alleviating role-related anxiety and role conflict (Karkkola et al., 2019). Accordingly, in the highly technologized and continuously evolving instructional environment of metaverse classroom, examining how teachers achieve role clarity through psychological adjustment mechanisms holds important theoretical and practical significance.

Drawing on awe experience theory, teachers confronted with instructional situations that exceed their prior teaching experiences often need to reinterpret and reposition their professional roles. Positive awe experiences provide psychological momentum for this process of role reconstruction by enhancing teachers’ perceptions of pedagogical possibilities and motivating professional growth. When teachers experience positive awe in metaverse classroom, they are more likely to approach new instructional demands with openness and an exploratory mindset, actively reflect on changes in their instructional responsibilities, and attempt to integrate emerging technologies into their teaching practices. Through this process, positive awe may facilitate role clarity by strengthening learning motivation and reducing psychological resistance to role change, thereby supporting teachers’ understanding and affirmation of their new instructional roles (Khalil and Jumani, 2024; Chiu, 2025). In contrast, negative awe experiences may influence teachers’ role perceptions through different psychological pathways. When teachers interpret technological complexity and instructional uncertainty as challenges that exceed their perceived capabilities, awe experiences may manifest as anxiety, diminished perceived control, or self-doubt (Keltner and Haidt, 2003). Such negative emotional experiences tend to undermine teachers’ self-efficacy and increase psychological resistance to role transformation, thereby impeding the development of clear role perceptions and potentially intensifying role ambiguity and uncertainty (Tarafdar et al., 2007). Based on these psychological mechanisms, the following hypotheses are proposed:

*H5a:* Positive awe experience significantly and positively influences teachers’ role clarity.

*H5b:* Negative awe experience significantly and negatively influences teachers’ role clarity.

### 2.4 Teaching engagement and teaching satisfaction

Role theory suggests that when individuals have a clear understanding of their responsibilities, expectations, and boundaries, their level of role clarity significantly increases (Lyons, 1971). Such clarity helps reduce role ambiguity and conflict, enabling teachers to allocate their attention and energy more effectively and to become more engaged in instructional activities (Farwa et al., 2025). Specifically, teaching engagement refers to teachers’ positive emotional and focused state during the teaching process. According to self-determination theory, this positive state arises when individuals experience a sense of autonomy, competence, and relatedness (Karkkola et al., 2019). Prior studies have confirmed that clear role recognition enhances teachers’ feelings of autonomy and competence, thereby fostering higher levels of work engagement (Granziera and Perera, 2019). At the same time, role clarity also contributes to teachers’ teaching satisfaction. Teaching satisfaction refers to the overall positive emotional evaluation that teachers hold toward their teaching experiences (Liu and Zhang, 2021). Clearly defined responsibilities and expectations can help reduce anxiety and uncertainty in new environments, strengthen teachers’ sense of control and confidence, and ultimately enhance their overall satisfaction with teaching. Therefore, the following hypotheses are proposed:

*H6:* Teachers’ role clarity significantly and positively influences teaching engagement.

*H7:* Teachers’ role clarity significantly and positively influences teaching satisfaction.

### 2.5 Model development

**Figure 1:**
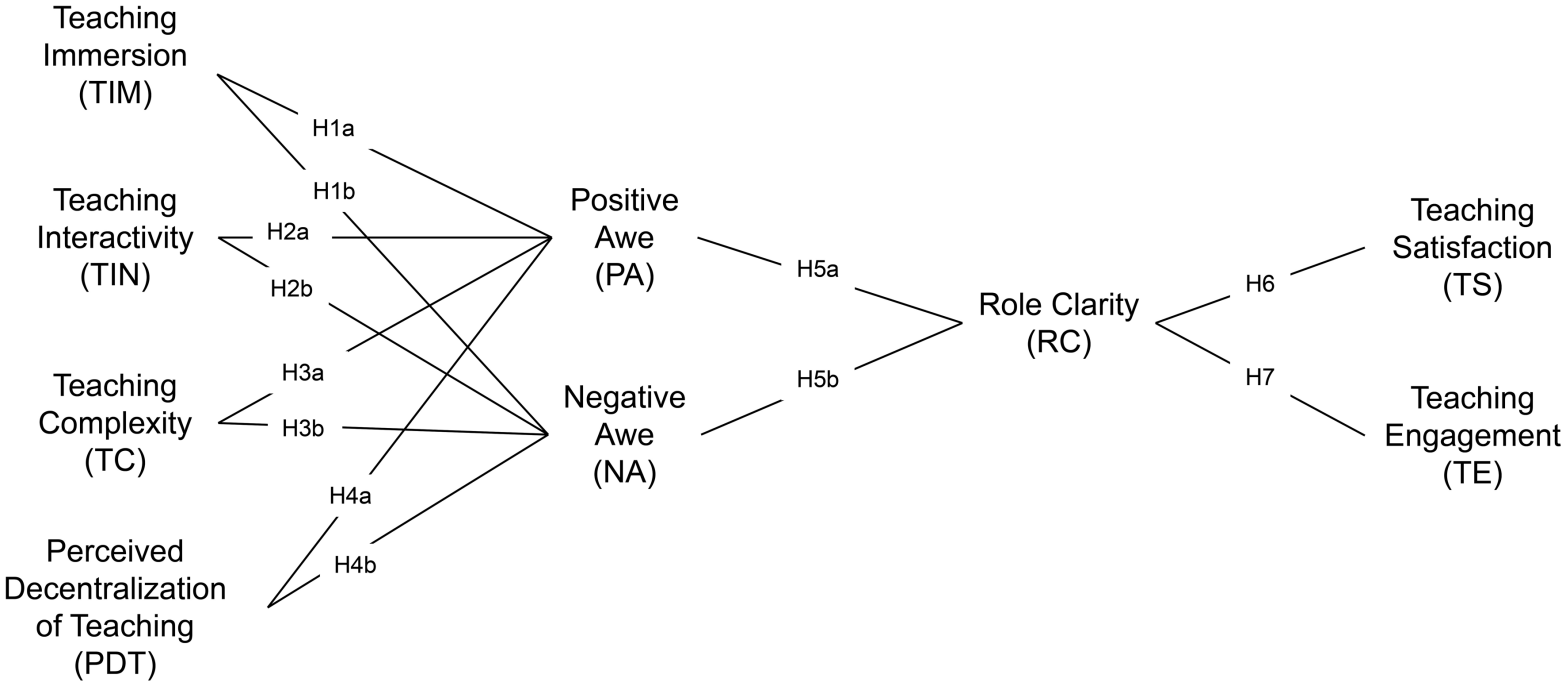
Research model.

Based on the above theoretical discussions and hypothesis development, this study proposes the research model shown in Figure 1. The model explores how features of the metaverse classroom influence teachers’ role clarity through their awe experiences, which in turn affect their teaching satisfaction and teaching engagement.

## 3 Method

### 3.1 Participants and data collection

This study adopted a stratified random sampling method, using the 184 primary and secondary schools designated as AI Education Demonstration Bases by the Chinese Ministry of Education in February 2024 as the sampling frame. These schools play a significant exemplary role in promoting the digital and intelligent transformation of basic education and are representative of teachers across different regions and educational stages in China with regard to their adaptation to metaverse classroom. The sampling procedure was as follows: First, the 184 schools were categorized into three levels based on educational stage primary schools, junior high schools, and senior high schools; then, approximately 200 teachers were randomly selected from each level to ensure a balanced distribution of participants across educational stages. It is important to note that the study targeted only frontline teachers who were actively involved in classroom teaching. Assistant teachers, administrative staff, technical support personnel, librarians, and staff responsible for student health and psychological support who were not directly engaged in classroom instruction were excluded from the sample. To reduce potential nonresponse bias, the research team clearly communicated the academic purpose of the study in the questionnaire invitation and ensured strict anonymity and confidentiality of participants’ information, thereby increasing their willingness to participate and improving the response rate.

The data for this study were collected through an online questionnaire survey. The survey consisted of the following four main sections: (1) A preliminary screening section asked participants whether they had actual teaching experience in metaverse classroom and whether they were responsible for concrete instructional tasks. If the answer was no, the survey automatically terminated to ensure data validity. (2) A research introduction and informed consent section explained the purpose of the study, emphasized the voluntary and anonymous nature of participation, stated that participants could withdraw at any time during the survey, and required participants to sign an informed consent form based on the Declaration of Helsinki. (3) A demographic information section collected basic information such as gender, teaching experience, and educational stage. (4) A measurement section included scales adopted from existing validated instruments, which were appropriately adapted to fit the context of this study to ensure content validity and practical applicability. Through this rigorous sampling design and data collection procedure, the study ensured a high degree of representativeness, reliability, and validity in the data, thereby providing a solid foundation for subsequent data analysis and theoretical testing.

The measurement instruments used in this study were adapted from well-established scales and moderately contextualized to fit the instructional setting of metaverse classroom. Teaching immersion was measured using three items adapted from Hwang et al. (2025), and teaching interactivity was assessed with four items adapted from Tan et al. (2019). Technical complexity was measured using three items adapted from Li and Wang (2021), while teaching decentralization was assessed with four items adapted from Turhan and Güneyli (2022). Awe experience was measured using eight items adapted from Zhao et al. (2025), comprising two dimensions: positive awe and negative awe. Teachers’ role clarity was measured with three items adapted from Hassan (2013). Teaching engagement was assessed using five items adapted from de Bruin and Henn (2013), and teaching satisfaction was measured with three items adapted from Liu and Zhang (2021). During the scale adaptation process, the original English items were first translated and back-translated to ensure semantic equivalence. Subsequently, selected items were contextually adjusted to reflect the instructional characteristics of metaverse classroom. For example, general references to teaching or technology use were explicitly specified as occurring within “metaverse classroom” or “immersive virtual teaching environments,” thereby enhancing contextual relevance. To ensure content validity, the adapted questionnaire was reviewed by five experts with backgrounds in educational technology or teaching practice. These experts evaluated the clarity, relevance, and appropriateness of the item wording, and minor revisions were made accordingly. The final questionnaire comprised 33 items covering nine latent variables. All items were measured using a five-point Likert scale ranging from 1 (“strongly disagree”) to 5 (“strongly agree”). The questionnaire was administered online via the Wenjuanxing platform, and participants completed the survey by scanning a QR code.

According to the Structural Equation Modeling sample size calculator (Soper, 2024), when setting the anticipated effect size at 0.3, the desired statistical power level at 0.9, the number of latent variables at 9, the number of observed variables at 33, and the probability level at 0.05, the minimum required sample size was determined to be 226. In this study, a total of 746 questionnaires were collected. After removing invalid responses, 692 valid questionnaires remained, resulting in an effective response rate of approximately 92.8%. This exceeds the minimum sample size requirement and meets the recommended criteria for SEM analysis (Hair et al., 2012).

### 3.2 Common method bias (CMB)

To examine whether common method bias existed in the data, Harman’s single-factor test was conducted. All measurement items were entered into an exploratory factor analysis using principal component extraction. The result showed that the unrotated first principal component accounted for 32.16 percent of the total variance, which is below the critical threshold of 40 percent (Podsakoff et al., 2003), indicating that common method bias was not a serious concern in this study.

In addition, variance inflation factor (VIF) values were calculated to assess potential multicollinearity issues among model variables (Shrestha, 2020). The analysis showed that all VIF values were below the recommended cutoff of 5, suggesting no significant multicollinearity and confirming good independence and discriminant validity among variables. Based on these results, this study ruled out the presence of serious common method bias and multicollinearity, ensuring the robustness and reliability of the subsequent data analysis.

## 4 Results

### 4.1 Demographic characteristics

The demographic characteristics of the participants are presented in Table 1. In terms of gender, the proportion of female teachers (59.8%) was significantly higher than that of male teachers (40.2%), which is consistent with the current gender distribution among teachers in China’s basic education system. Regarding teaching experience, the sample was relatively young, with the largest group being teachers with 6–10 years of experience (31.5%), followed by those with 5 years or less (27.7%), indicating a younger demographic among teachers involved in metaverse classroom teaching. As for the teaching stages, the sample was evenly distributed across primary, junior high, and senior high school levels, accounting for 32.9, 34.0, and 33.1%, respectively. This balance ensures that the sample reflects teachers’ adaptation and experience differences across various educational stages in metaverse classroom. In terms of educational background, 84.9% of the participants held a bachelor’s degree or above, among whom 38.4% had a postgraduate degree, indicating that the teachers involved in metaverse classroom teaching generally possessed a high level of educational attainment. Regarding usage frequency, 71.4% of teachers reported using metaverse classroom at least once per week, suggesting a relatively high level of engagement. Among them, the highest proportion used the metaverse classroom once a week, followed by those who used it more than once a week, showing that a considerable number of teachers have integrated metaverse classroom into their regular teaching practices and demonstrated strong technology acceptance and usage motivation.

**Table 1:** Demographic characteristics of the participants.

Demographic information	Category	Frequency	Percentage (%)
Gender	Male	278	40.2
	Female	414	59.8
Teaching age	5 years or less	192	27.7
	6–10 years	218	31.5
	11–15 years	165	23.8
	16–20 years	75	10.8
	More than 21 years	42	6.1
Teaching grade	Primary School	228	32.9
	Junior High School	235	34.0
	Senior High School	229	33.1
Highest educational qualification	Associate degree or below	104	15.0
	Bachelor’s degree	322	46.5
	Postgraduate or above	266	38.4
Frequency of use	Once per month	82	11.8
	Once every 2 weeks	116	16.8
	Once per week	218	31.5
	More than once per week	157	22.7
	Once per day	74	10.7
	More than once per day	45	6.5

Overall, the demographic characteristics of the sample indicate good representativeness and alignment with the current profile of metaverse classroom teachers, providing a solid foundation for subsequent analysis.

### 4.2 Reliability, validity, and factor loadings

This study employed partial least squares structural equation modeling (PLS-SEM) and used SmartPLS 4.0 to evaluate the reliability and validity of the measurement model. Following the recommendations of Sarstedt et al. (2017), multiple indicators were used to assess internal consistency and validity, including Cronbach’s alpha, rho_A, composite reliability (CR), and average variance extracted (AVE).

As shown in Table 2, the Cronbach’s alpha values for all latent variables ranged from 0.796 to 0.917, exceeding the recommended threshold of 0.7. In addition, the rho_A and CR values also surpassed the standard cutoff of 0.7, indicating high internal consistency and strong reliability of the measurement instruments (Christmann and Van Aelst, 2006). AVE values were used to assess convergent validity. All latent constructs had AVE values between 0.685 and 0.800, which are well above the recommended threshold of 0.5, demonstrating good convergent validity for all constructs in this study.

**Table 2:** Reliability and AVE values.

Constructs	Cronbach’s alpha	rho_a	rho_c	AVE
NA	0.917	0.917	0.941	0.800
PA	0.885	0.885	0.920	0.743
PDT	0.847	0.848	0.897	0.685
RC	0.846	0.847	0.907	0.764
TC	0.796	0.796	0.880	0.710
TE	0.828	0.828	0.897	0.744
TIM	0.843	0.844	0.906	0.762
TIN	0.859	0.859	0.904	0.703
TS	0.891	0.891	0.920	0.696

As shown in Table 3, all factor loadings of the measurement items in this study ranged from 0.813 to 0.901, exceeding the recommended threshold of 0.7 (Christmann and Van Aelst, 2006). These results indicate that all items have strong loadings on their respective latent constructs, demonstrating high explanatory power and confirming the overall validity of the measurement model.

**Table 3:** Factor loadings.

Indicator	NA	PA	PDT	RC	TC	TE	TIM	TIN	TS	VIF
NA1	0.901									3.016
NA2	0.895									2.870
NA3	0.890									2.815
NA4	0.891									2.878
PA1		0.865								2.319
PA2		0.858								2.187
PA3		0.861								2.301
PA4		0.862								2.295
PDT1			0.828							1.896
PDT2			0.841							1.977
PDT3			0.829							1.861
PDT4			0.813							1.840
RC1				0.887						2.127
RC2				0.873						2.037
RC3				0.863						1.953
TC1					0.854					1.749
TC2					0.843					1.690
TC3					0.831					1.638
TE1						0.860				1.854
TE2						0.858				1.864
TE3						0.869				1.951
TIM1							0.881			2.076
TIM2							0.858			1.896
TIM3							0.879			2.093
TIN1								0.845		2.039
TIN2								0.844		2.041
TIN3								0.824		1.908
TIN4								0.839		1.996
TS1									0.833	2.158
TS2									0.826	2.105
TS3									0.833	2.186
TS4									0.845	2.244
TS5									0.835	2.183

Discriminant validity was assessed using both the Fornell–Larcker criterion and the Heterotrait–Monotrait (HTMT) ratio. As shown in Table 4, according to the Fornell–Larcker standard (Fornell and Larcker, 1981), the square root of the AVE for each latent construct was greater than its correlations with any other constructs. This indicates that the measurement model has satisfactory discriminant validity.

**Table 4:** Fornell–Larcker.

Constructs	NA	PA	PDT	RC	TC	TE	TIM	TIN	TS
NA	0.894								
PA	−0.398	0.862							
PDT	−0.506	0.451	0.828						
RC	−0.378	0.343	0.398	0.874					
TC	−0.498	0.416	0.426	0.370	0.843				
TE	−0.313	0.317	0.406	0.505	0.314	0.863			
TIM	−0.498	0.511	0.413	0.342	0.363	0.297	0.873		
TIN	−0.477	0.494	0.421	0.335	0.329	0.351	0.441	0.838	
TS	−0.369	0.347	0.405	0.485	0.365	0.442	0.365	0.314	0.834

In addition, as shown in Table 5, all HTMT values ranged from 0.359 to 0.603, which are below the recommended threshold of 0.85 (Hair et al., 2019), further confirming adequate discriminant validity. These results suggest that the measurement instruments can effectively distinguish between different constructs.

**Table 5:** HTMT.

Constructs	NA	PA	PDT	RC	TC	TE	TIM	TIN	TS
NA									
PA	0.442								
PDT	0.573	0.519							
RC	0.429	0.397	0.470						
TC	0.583	0.495	0.518	0.451					
TE	0.359	0.370	0.485	0.603	0.387				
TIM	0.566	0.591	0.488	0.405	0.443	0.356			
TIN	0.537	0.566	0.493	0.393	0.398	0.416	0.518		
TS	0.408	0.391	0.465	0.558	0.433	0.514	0.421	0.359	

### 4.3 Model fit, explanatory power, and predictive relevance

To evaluate the model’s quality of fit and predictive relevance, this study assessed the structural model using *R*^2^ and *Q*^2^ statistics (Shmueli and Koppius, 2011). As shown in Table 6, the *R*^2^ values of the endogenous latent variables ranged from 0.187 to 0.447, all well above zero, indicating a good level of explanatory power for the structural model. The adjusted *R*^2^ values ranged from 0.185 to 0.444, also demonstrating strong model robustness and explanatory performance. In addition, *Q*^2^ values were calculated to further assess the model’s predictive relevance. As presented in Table 6, all *Q*^2^ values were greater than zero, ranging from 0.142 to 0.354, suggesting that the model has strong predictive capability. These results indicate that the proposed structural paths can effectively support predictions related to teachers’ role adaptation and teaching outcomes in metaverse classroom settings.

**Table 6:** *R*^2^ and *Q*^2^.

Variable	*R* ^2^	Adjusted *R*^2^	*Q*^2^(=1-SSE/SSO)
NA	0.447	0.444	0.354
PA	0.405	0.402	0.296
RC	0.187	0.185	0.142
TE	0.255	0.253	0.188
TS	0.235	0.234	0.162

In addition, as shown in Table 7, the standardized root mean square residual (SRMR) value of the model was 0.035, which is below the recommended threshold of 0.08, indicating a good fit between the data and the theoretical model (Henseler et al., 2015). The normed fit index (NFI) value was 0.885, exceeding the recommended benchmark of 0.8, further demonstrating that the proposed model exhibits high overall model fit quality.

**Table 7:** SRMR and NFI.

Model fit index	Value
SRMR	0.035
NFI	0.885

### 4.4 Path coefficient analysis

The results of the path coefficient analysis are presented in Table 8. Regarding the effects of classroom characteristics on awe experience, perceived decentralization of teaching (PDT) had a significant negative effect on negative awe (NA) (*β* = −0.219, *p* < 0.05) and a significant positive effect on positive awe (PA) (*β* = 0.162, *p* < 0.05). Technical complexity (TC) negatively influenced NA (*β* = −0.257, *p* < 0.05) and positively influenced PA (*β* = 0.165, *p* < 0.05). Teaching immersion (TIM) showed a significant negative effect on NA (*β* = −0.226, *p* < 0.05) and a significant positive effect on PA (*β* = 0.274, *p* < 0.05). Teaching interactivity (TIN) also had a significant negative impact on NA (*β* = −0.201, *p* < 0.05) and a significant positive impact on PA (*β* = 0.251, *p* < 0.05).

**Table 8:** Path coefficient results.

Path	Original sample	STDEV	T-value	*p*-value	Hypothesis support
NA → RC	−0.287	0.038	7.624	0.000	Yes
PA → RC	0.229	0.041	5.579	0.000	Yes
PDT → NA	−0.219	0.040	5.501	0.000	No
PDT → PA	0.162	0.042	3.888	0.000	Yes
RC → TE	0.505	0.034	14.686	0.000	Yes
RC → TS	0.485	0.035	13.984	0.000	Yes
TC → NA	−0.257	0.036	7.138	0.000	No
TC → PA	0.165	0.037	4.398	0.000	Yes
TIM → NA	−0.226	0.039	5.822	0.000	No
TIM → PA	0.274	0.041	6.628	0.000	Yes
TIN → NA	−0.201	0.037	5.402	0.000	No
TIN → PA	0.251	0.041	6.079	0.000	Yes

For the effects of awe experience on role adaptation, PA had a significant positive effect on role clarity (RC) (*β* = 0.229, *p* < 0.05), while NA had a significant negative effect on RC (*β* = −0.287, *p* < 0.05).

Regarding the effects of role clarity on teaching outcome variables, RC significantly and positively influenced both teaching engagement (TE) (*β* = 0.505, *p* < 0.05) and teaching satisfaction (TS) (*β* = 0.485, *p* < 0.05).

**Figure 2:**
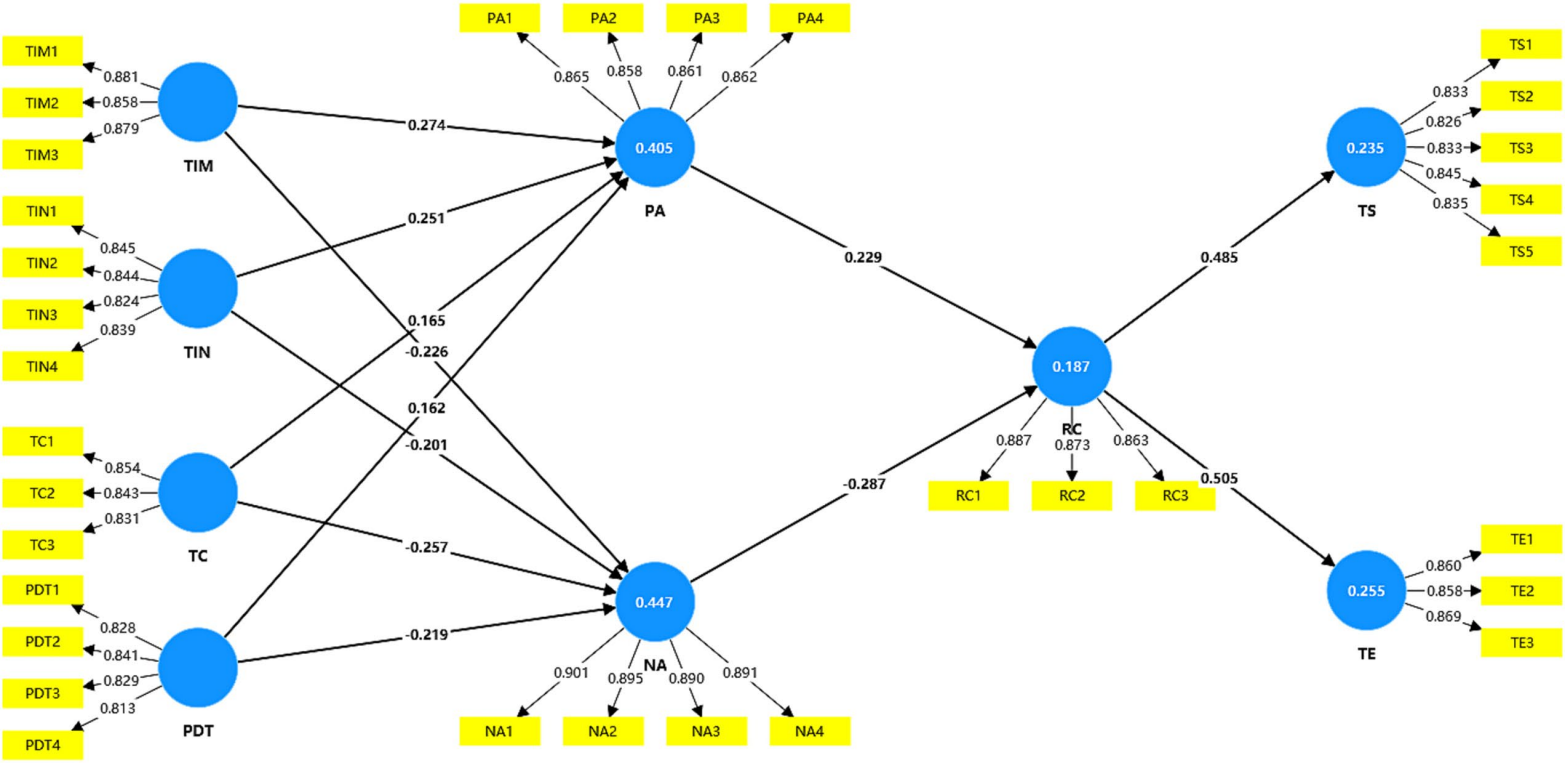
Structural model of the hypothesized relationships.

The structural equation model of the relationships among variables is presented in Figure 2.

### 4.5 Multi-group analysis

To further explore potential differences in path coefficients across teachers from different educational stages, a multi-group analysis (MGA) was conducted. Before performing group comparisons, it was necessary to ensure measurement invariance across groups. According to prior research and established guidelines (Cheah et al., 2023), the MICOM (Measurement Invariance of Composite Models) procedure involves three steps: (1) configural invariance, (2) compositional invariance, (3) equality of means and variances.

Using the permutation-based MICOM procedure in PLS-SEM, this study confirmed that measurement invariance was established across groups (see Table 9). Therefore, group comparisons could be validly performed.

**Table 9:** Measurement invariance assessment via MICOM.

Group	Con.	Conf. Inv.	Comp. Inv.		PMI Est.	Equal Mean		Equal Var		FMI Est.
			C = 1	CI.		Diff.	CI.	Diff.	CI.	
Primary vs. Junior High	NA	Yes	1.000	[1.000; 1.000]	Yes	0.366	[−0.175; 0.191]	0.116	[−0.178; 0.176]	No
	PA	Yes	1.000	[0.999; 1.000]	Yes	−0.127	[−0.193; 0.174]	0.163	[−0.210; 0.206]	Yes
	PDT	Yes	0.999	[0.998; 1.000]	Yes	−0.129	[−0.178; 0.174]	0.061	[−0.187; 0.184]	Yes
	RC	Yes	1.000	[0.999; 1.000]	Yes	−0.298	[−0.186; 0.184]	0.166	[−0.211; 0.193]	No
	TC	Yes	1.000	[0.997; 1.000]	Yes	−0.242	[−0.183; 0.171]	0.227	[−0.200; 0.182]	No
	TE	Yes	1.000	[0.998; 1.000]	Yes	−0.088	[−0.177; 0.176]	−0.010	[−0.221; 0.207]	Yes
	TIM	Yes	1.000	[0.999; 1.000]	Yes	−0.146	[−0.178; 0.187]	0.004	[−0.245; 0.241]	Yes
	TIN	Yes	0.999	[0.999; 1.000]	Yes	−0.246	[−0.182; 0.174]	0.352	[−0.228; 0.216]	No
	TS	Yes	0.998	[0.998; 1.000]	Yes	−0.046	[−0.192; 0.169]	−0.078	[−0.210; 0.225]	Yes
Junior High vs. Senior High	NA	Yes	1.000	[1.000; 1.000]	Yes	−0.265	[−0.187; 0.176]	−0.311	[−0.170; 0.180]	No
	PA	Yes	0.999	[0.999; 1.000]	Yes	−0.050	[−0.181; 0.192]	0.117	[−0.237; 0.230]	Yes
	PDT	Yes	0.998	[0.998; 1.000]	Yes	0.058	[−0.177; 0.181]	−0.082	[−0.191; 0.184]	Yes
	RC	Yes	1.000	[0.999; 1.000]	Yes	0.201	[−0.179; 0.188]	−0.097	[−0.218; 0.202]	No
	TC	Yes	0.999	[0.997; 1.000]	Yes	0.112	[−0.193; 0.174]	−0.092	[−0.235; 0.224]	Yes
	TE	Yes	1.000	[0.998; 1.000]	Yes	−0.001	[−0.176; 0.191]	0.091	[−0.222; 0.235]	Yes
	TIM	Yes	1.000	[0.999; 1.000]	Yes	0.049	[−0.189; 0.180]	0.078	[−0.239; 0.265]	Yes
	TIN	Yes	0.997	[0.997; 1.000]	Yes	0.112	[−0.180; 0.187]	−0.195	[−0.237; 0.249]	Yes
	TS	Yes	1.000	[0.998; 1.000]	Yes	0.076	[−0.183; 0.177]	−0.079	[−0.202; 0.208]	Yes
Primary vs. Senior High	NA	Yes	1.000	[1.000; 1.000]	Yes	0.085	[−0.172; 0.163]	−0.192	[−0.154; 0.164]	No
	PA	Yes	1.000	[0.999; 1.000]	Yes	−0.177	[−0.164; 0.162]	0.281	[−0.210; 0.207]	No
	PDT	Yes	0.999	[0.998; 1.000]	Yes	−0.075	[−0.167; 0.182]	−0.024	[−0.187; 0.207]	Yes
	RC	Yes	1.000	[0.999; 1.000]	Yes	−0.103	[−0.171; 0.176]	0.068	[−0.166; 0.174]	Yes
	TC	Yes	1.000	[0.998; 1.000]	Yes	−0.132	[−0.163; 0.175]	0.142	[−0.185; 0.194]	Yes
	TE	Yes	1.000	[0.997; 1.000]	Yes	−0.093	[−0.163; 0.157]	0.074	[−0.212; 0.229]	Yes
	TIM	Yes	1.000	[0.999; 1.000]	Yes	−0.101	[−0.163; 0.164]	0.085	[−0.235; 0.255]	Yes
	TIN	Yes	0.999	[0.999; 1.000]	Yes	−0.133	[−0.160; 0.166]	0.162	[−0.219; 0.238]	Yes
	TS	Yes	0.999	[0.998; 1.000]	Yes	0.031	[−0.173; 0.181]	−0.152	[−0.221; 0.204]	Yes

Con., Construct; Conf. Inv., Configural Invariance; Comp. Inv., Compositional Invariance; CI, Confidence Interval; PMI Est., Partial Measurement Invariance Established; Equal Mean, Equal Mean Value; Diff., Differences; Equal Var., Equal Variances; FMI Est., Full Measurement Invariance Established.

To further uncover structural differences in path relationships across teachers from different educational stages, MGA was conducted following the MICOM measurement invariance test. The PLS-MGA results for paths with significant differences are presented in Table 10.

**Table 10:** Multi-group analysis results.

Group	Relationship	β0	β1	Coefficient difference
Junior High (0) VS Primary (1)	TIM → PA	0.180**	0.383***	0.203*
	TIN→PA	0.149*	0.367***	0.218*
Junior High (0) VS Senior High (1)	TC → NA	−0.207**	0.403***	0.196*
	TIM → NA	−0.166*	−0.393***	0.227*
Primary (0) VS Senior High (1)	TC → NA	−0.174**	−0.403***	0.229**
	TIM → NA	−0.150*	0.393***	0.243**

*p < 0.05; **p < 0.01; ***p < 0.001.

Specifically, the paths from teaching immersion (TIM) to positive awe (PA) and from teaching interactivity (TIN) to PA showed significantly higher path coefficients among primary school teachers compared to junior high school teachers. This suggests that primary school teachers are more emotionally responsive to immersive and interactive classroom environments. In the path from technical complexity (TC) to negative awe (NA), significant differences were observed between junior high and senior high teachers, as well as between primary and senior high teachers. These findings imply that senior high school teachers possess stronger emotional regulation when confronted with complex technologies. Additionally, the TIM → NA path also showed significant differences between junior high and senior high, and between primary and senior high groups, further confirming that senior high school teachers are better adapted to high-immersion environments.

Overall, the multi-group analysis results reveal significant differences in emotional responses to the metaverse classroom across educational stages, providing empirical support for stage-specific interventions and targeted teacher training strategies.

## 5 Discussion

First, this study systematically examined how teaching immersion, teaching interactivity, technical complexity, and perceived decentralization in metaverse classroom influence teachers’ role-related outcomes through positive and negative awe experiences (H1–H4). The results indicate that all four characteristics of metaverse classroom significantly enhanced teachers’ positive awe experiences, providing support for hypotheses H1a, H2a, H3a, and H4a. This finding suggests that the immersive, interactive, and technologically enriched instructional environments fostered by metaverse classroom are effective in eliciting positive emotional experiences among teachers, which is consistent with prior research on immersive technologies and teachers’ emotional responses (Chirico et al., 2017; Quesnel and Riecke, 2018; Kahn and Cargile, 2021; Zhao et al., 2025).

However, with respect to negative awe experiences, the results revealed a pattern that differed from the initial hypotheses. Specifically, the aforementioned characteristics of metaverse classroom were found to significantly reduce teachers’ negative awe experiences overall, and thus hypotheses H1b, H2b, H3b, and H4b were not supported. This finding indicates that, within the context of metaverse classroom, these technological and environmental features do not exacerbate teachers’ negative emotional responses as initially expected. Instead, they may alleviate teachers’ discomfort through environmental support and emotion regulation mechanisms. This result offers a new perspective for understanding teachers’ emotional adaptation processes in emerging technology-enhanced teaching environments.

More specifically, teaching immersion appears to reduce anxiety and uncertainty arising from technological unfamiliarity by providing highly realistic and coherent instructional scenarios that facilitate teachers’ rapid understanding and adaptation to the technological environment (Al-kfairy et al., 2024). Similarly, teaching interactivity—through real-time feedback mechanisms and multi-agent interaction formats—enables teachers to adjust their instructional behaviors in a timely manner and to receive support from both the system and peers during the teaching process, thereby alleviating feelings of isolation and emotional unease (Arghashi, 2024; Kiu and Lam, 2024). In addition, perceived decentralization may help disperse the pressure associated with teachers’ sole control over the classroom. By redistributing instructional responsibilities and interactional authority, decentralization can reduce teachers’ emotional load in complex teaching situations and contribute to the attenuation of negative emotional responses (Zhu, 2024). Taken together, these findings suggest that metaverse classroom, as a whole, provide a supportive instructional environment that helps mitigate negative emotional experiences during the initial stages of technology use.

Of particular interest, technical complexity was also found to exert a significant suppressive effect on teachers’ negative awe experiences. At first glance, this result appears to diverge from prior research that conceptualizes technical complexity primarily as a source of cognitive burden (Brown, 2015). However, this divergence can be theoretically explained by the different psychological levels at which these constructs operate. Brown (2015) conceptualizes technical complexity mainly as a source of cognitive load, emphasizing increased information-processing demands and mental effort during task execution. In contrast, the present study focuses on teachers’ awe-related emotional responses while working within metaverse classroom environments. Cognitive load primarily reflects momentary processing pressure, whereas awe experience captures a more holistic emotional response to perceived vastness and the accompanying need for cognitive accommodation. Although these two constructs are related at the experiential level, they operate through distinct psychological mechanisms. Within metaverse classroom, technical complexity is not limited to operational difficulty but also reflects functional complexity associated with the integration of instructional features and the reconstruction of teaching practices. When teachers interpret such complexity as a resource that supports instructional innovation and professional development, the associated challenge is more likely to be transformed into an adjustable teaching task rather than an overwhelming or uncontrollable emotional experience. Under these conditions, technical complexity does not directly intensify negative awe; instead, it may reduce feelings of loss of control by activating teachers’ professional coping awareness and instructional strategy adjustment, thereby suppressing negative awe experiences. This finding suggests that, given clear instructional goals and sufficient practical experience, technical complexity may be more likely to elicit adaptive emotional responses among teachers rather than functioning solely as an emotional burden.

Second, focusing on hypothesis H5a, this study further examined the role of positive and negative awe experiences in teachers’ role adaptation processes. The results indicate that positive awe experience has a significant positive effect on teachers’ role clarity, thereby supporting hypothesis H5a. This finding suggests that when teachers experience a sense of inspiration and wonder triggered by new technologies, they are more likely to embrace role transformation, demonstrating greater proactivity and openness. In contrast, negative awe significantly hindered teachers’ role adaptation, which aligns with the theoretical perspective of threat-based awe (Gordon et al., 2017). When teachers perceive the technological environment as beyond their control, they are prone to psychological resistance and avoidance behaviors, thereby reducing their willingness to adapt to new roles. Such negative emotional states become a major barrier to effective role adaptation. These findings suggest that future teacher training and educational technology development should pay particular attention to mitigating negative awe while enhancing positive awe, in order to facilitate teachers’ smooth transition into new instructional roles.

Third, this study confirms the critical role of teachers’ role clarity in subsequent teaching-related outcomes. The results show that role clarity has a significant positive effect on both teaching satisfaction and teaching engagement, thereby supporting hypotheses H6 and H7. Role clarity contributes to strengthening teachers’ sense of self-efficacy and professional identity, enabling them to experience greater intrinsic value and a sense of achievement in technology-enhanced environments (Wu and Santos, 2024). When teachers have a clear understanding of their roles and responsibilities, they are more inclined to explore and apply innovative classroom features, demonstrating higher levels of teaching enthusiasm and creativity (Huang et al., 2019). This finding highlights that role clarity and adaptation are not merely psychological processes but are directly linked to teaching behaviors and instructional performance. Therefore, in the implementation of educational technologies, it is essential to provide sufficient psychological support and role adaptation training, in order to comprehensively improve teaching quality and support teachers’ long-term professional development.

Finally, based on the results of the multi-group analysis, significant differences were observed across educational stages in teachers’ emotional response pathways. First, primary school teachers differed significantly from junior high school teachers in the path to positive awe. Specifically, in the path from teaching immersion (TIM) to positive awe (PA), primary teachers (*β* = 0.383) exhibited a much stronger effect than junior high school teachers (*β* = 0.180). Similarly, in the path from teaching interactivity (TIN) to PA, primary teachers (*β* = 0.367) also showed significantly higher responsiveness than their junior high counterparts (*β* = 0.149). These results suggest that primary school teachers are more emotionally receptive to immersive and interactive experiences, demonstrating stronger psychological adaptability and a faster acceptance of role transformation in the metaverse classroom. Second, clear differences were observed between high school and middle school teachers in the pathways associated with negative awe experiences, reflecting that teachers at different educational stages exhibit distinct emotional response patterns when confronting technological characteristics of metaverse classroom. Specifically, in the path from technical complexity (TC) to negative awe (NA), the high school teacher group demonstrated a significant positive effect (*β* = 0.403), indicating that as technical complexity increases, high school teachers are more likely to interpret complexity as a challenging emotional stimulus, thereby eliciting stronger negative awe responses. In contrast, middle school teachers exhibited a significant negative effect on this path (*β* = −0.207), suggesting a greater tendency to regulate and mitigate negative emotional experiences as technical complexity increases. In addition, for the path from teaching immersion (TIM) to negative awe (NA), high school teachers showed a stronger negative effect (*β* = −0.393), indicating that immersive instructional environments play a more pronounced role in alleviating negative awe experiences among high school teachers. This result suggests that high school teachers may exhibit different patterns of emotional interpretation and regulation in immersive teaching contexts, rather than differences attributable solely to technological capability. It should be emphasized that these observed differences reflect variations in emotional response pathways and psychological adaptation patterns, rather than direct judgments regarding teachers’ levels of technical training, usage frequency, or problem-solving abilities. Such factors warrant further investigation through more fine-grained measurements in future research.

The above findings and analysis reveal that teachers at different educational stages exhibit distinct emotional responses and technology adaptation patterns. When considered in relation to the practical teaching context, primary school teachers generally face lighter teaching responsibilities and lower subject complexity, while receiving more lively and positive feedback from students. As a result, they demonstrate stronger experiences of positive emotions in the metaverse classroom and are more easily influenced by immersive and interactive teaching features. In contrast, high school teachers are subject to greater academic pressure and more complex teaching tasks. Although they may experience higher cognitive load and anxiety due to technological complexity, this complexity can be perceived positively. Given that high school curricula often involve abstract and conceptually challenging subjects such as physics and chemistry, metaverse technologies can help visualize and concretize abstract knowledge, thereby easing instructional difficulties. In this context, high school teachers demonstrate stronger emotional adaptability to technologically complex environments, showing a greater tendency to interpret complexity as manageable instructional challenges rather than overwhelming stressors, which in turn enables them to better regulate the negative emotional effects of technical complexity. By comparison, junior high school teachers face a moderate level of instructional demands. Their students experience more academic pressure than those in primary school but less than those in high school, resulting in a relatively balanced emotional and adaptive profile. However, their responses across most indicators remain lower than the more extreme reactions observed in the other two groups, reflecting an overall intermediate state.

In summary, future educational technology development and teacher training programs should carefully consider the emotional and technological adaptation differences across primary, junior high, and high school teachers. Tailored psychological and technological support should be provided to enhance effective adaptation and promote active engagement among teachers at all educational levels.

## 6 Conclusion

### 6.1 Theoretical contributions

At the theoretical level, this study systematically integrates the characteristics of metaverse classroom with teachers’ awe experiences, constructing an overall explanatory pathway that links instructional environment features, emotional experiences, teachers’ role cognition, and subsequent teaching-related behaviors. Departing from the traditional stress-oriented perspective, the findings reveal the complexity and dual nature of awe, offering a new emotional lens for educational technology research. In addition, through multi-group analysis, the study refines our understanding of teachers’ emotional responses and adaptation strategies across educational stages, providing a more precise theoretical foundation for future technology development and teacher training in metaverse classroom. Specifically, the study contributes to the literature in the following ways:

First, with reference to hypotheses H1–H4, this study systematically examines the mechanisms through which metaverse classroom characteristics influence teachers’ awe experiences, drawing on an integrative perspective that combines emotional psychology and educational technology. The findings indicate that teaching immersion and teaching interactivity in metaverse classroom significantly enhance teachers’ positive awe experiences, while not reinforcing negative awe experiences at an overall level. This result extends prior research that has predominantly conceptualized emerging technologies as sources of teacher stress, suggesting instead that, within metaverse classroom contexts, technological environment features may help teachers reinterpret and cope with instructional complexity by eliciting positive emotional experiences and facilitating emotion regulation mechanisms.

Second, with reference to hypothesis H5, this study introduces awe experience as a complex emotional mechanism into the research framework of teachers’ role clarity, thereby extending the theoretical boundaries of research on teacher emotions and role adaptation. Previous studies have typically adopted a stress-centered or single-emotion perspective to explain how teachers adapt to emerging technologies. In contrast, this study emphasizes the multidimensional nature of emotions that teachers experience in the metaverse classroom, including both positive emotional expansion and inspiration, as well as the stress and challenges triggered by unfamiliar contexts and complex technologies. This comprehensive emotional perspective breaks away from traditional unidimensional models and helps construct a more nuanced and diversified theoretical framework of emotional adaptation among teachers.

Third, through systematic multi-group analysis, this study offers a pioneering investigation into the differentiated emotional responses and adaptation patterns among primary, junior high, and high school teachers in the metaverse classroom. The results reveal that primary school teachers are more readily motivated by positive emotional cues, high school teachers exhibit stronger technological adaptability and emotional regulation in response to challenges, while junior high school teachers remain in a relatively balanced intermediate state. These stage-specific differences contribute to a clearer theoretical framework for teacher adaptability and provide a concrete foundation for future investigations into role transformation and psychological adjustment under emerging technological conditions.

In summary, this study significantly expands the theoretical boundaries of research on teacher adaptation, emotional psychology, and role theory through conceptual integration and innovation. The findings deepen the understanding of teachers’ role transformation and psychological mechanisms of adaptation in technology-enhanced educational environments and offer a solid theoretical foundation and forward-looking reference for future research and educational practice.

### 6.2 Practical implications

The practical contributions of this study primarily lie in providing empirically grounded reference directions for the design of metaverse classroom, teachers’ instructional practice, and teacher support strategies, with the aim of facilitating teachers’ emotional adaptation and role adjustment in emerging instructional environments.

First, at the level of metaverse classroom design, the findings indicate that teaching immersion and teaching interactivity contribute to the enhancement of teachers’ positive awe experiences while, at an overall level, alleviating negative emotional responses. This suggests that metaverse classroom platforms may benefit from prioritizing immersive scene construction and the optimization of interaction mechanisms, thereby strengthening teachers’ emotional acceptance of the instructional environment. At the same time, technical complexity and perceived decentralization do not necessarily intensify teachers’ negative emotions; rather, their effects depend on how teachers interpret technological functions and instructional roles. Accordingly, when technical complexity is unavoidable, platform design may place greater emphasis on clear functional presentation and instructional support to reduce teachers’ uncertainty during the initial stages of use.

Second, from the perspective of frontline teachers, this study highlights the importance of emotional experiences in the process of role adaptation. The results show that positive awe experiences are associated with higher levels of role clarity, which in turn contribute to greater teaching engagement and teaching satisfaction. This finding suggests that, during participation in metaverse classroom teaching, teachers may benefit from attending to how their emotional experiences shape their understanding and positioning of instructional roles, and from gradually developing stable role perceptions that support adaptation to new teaching environments.

Finally, from the perspective of educational management and teacher support, this study reveals that teachers at different educational stages exhibit distinct emotional response pathways in metaverse classroom. This finding indicates that teacher training and support strategies may need to take teaching stage differences into account, rather than relying on uniform approaches to technology implementation or support. Providing support that is aligned with instructional contexts and teachers’ needs may help facilitate teachers’ sustained participation and emotional adaptation in metaverse classroom settings.

In summary, this study provides empirical evidence for understanding teachers’ emotional experiences and role adaptation in metaverse classroom, and offers cautious yet practical reference directions for related educational practices.

### 6.3 Limitations

First, as metaverse classroom teaching is still in the early stages of promotion and exploration, the population of teachers who have actually participated in or experienced this instructional model remains relatively limited. Consequently, the sample in this study was drawn primarily from a specific group of teachers with relevant experience. This sampling constraint may limit the generalizability and external validity of the findings. Future studies should aim to expand the sampling scope as the adoption of metaverse classroom increases, thereby enhancing the representativeness and robustness of the results.

Second, although this study focuses on the emotional mechanisms linking awe experience to teachers’ role clarity in metaverse classroom, several potentially relevant factors were not explicitly incorporated into the proposed model. For example, teachers’ prior experience with educational technologies, institutional support for metaverse implementation, and levels of digital literacy may all influence both awe experiences and role clarity. Future research may integrate these factors to further disentangle their respective roles and to examine how emotional mechanisms interact with contextual and individual differences in shaping teachers’ role adaptation processes.

Third, although this study considered group differences across educational stages, it did not further differentiate teachers based on subject area, years of teaching experience, or individual characteristics such as personality traits or technology acceptance levels. These individual-level factors likely play a role in how teachers adapt to metaverse classroom and may influence their emotional and behavioral responses. Future research could incorporate these variables to provide a more comprehensive and nuanced understanding of the individual differences and complex mechanisms underlying role adaptation in technology-enhanced learning environments.

Finally, the study employed a cross-sectional research design, capturing teachers’ awe experiences and role adaptation at a single point in time. Given that both awe and role clarity are dynamic psychological processes, this design limits our ability to understand how these constructs evolve over time. Future studies are encouraged to adopt longitudinal designs to explore the temporal progression of teachers’ emotional experiences and adaptive responses. Moreover, the inclusion of physiological measurements or scenario-based experiments could yield more objective insights into teachers’ emotional states and adaptation processes in immersive virtual learning environments.

## Data Availability

The original contributions presented in the study are included in the article/supplementary material, further inquiries can be directed to the corresponding author.
